# Lapatinib inhibits CIP2A/PP2A/p-Akt signaling and induces apoptosis in triple negative breast cancer cells

**DOI:** 10.18632/oncotarget.7035

**Published:** 2016-01-27

**Authors:** Chun-Yu Liu, Ming-Hung Hu, Chia-Jung Hsu, Chun-Teng Huang, Duen-Shian Wang, Wen-Chun Tsai, Yi-Ting Chen, Chia-Han Lee, Pei-Yi Chu, Chia-Chi Hsu, Ming-Huang Chen, Chung-Wai Shiau, Ling-Ming Tseng, Kuen-Feng Chen

**Affiliations:** ^1^ Division of Medical Oncology, Department of Oncology, Taipei Veterans General Hospital, Taipei, Taiwan; ^2^ School of Medicine, National Yang-Ming University, Taipei, Taiwan; ^3^ Division of Hematology and Oncology, Department of Medicine, Cardinal Tien Hospital, New Taipei City, Taiwan; ^4^ School of Medicine, Fu Jen Catholic University, New Taipei City, Taiwan; ^5^ Division of Hematology & Oncology, Department of Medicine, Yang-Ming Branch of Taipei City Hospital, Taipei, Taiwan; ^6^ Institute of Biopharmaceutical Sciences, National Yang-Ming University, Taipei, Taiwan; ^7^ Department of Surgery, Taipei Veterans General Hospital, Taipei, Taiwan; ^8^ Department of Medical Research, National Taiwan University Hospital, Taipei, Taiwan; ^9^ National Taiwan University College of Medicine, Taipei, Taiwan; ^10^ Department of Pathology, Show Chwan Memorial Hospital, Changhua City, Taiwan; ^11^ Institute of Pharmacology, National Yang-Ming University, Taipei, Taiwan

**Keywords:** lapatinib, triple-negative breast cancer, PP2A, CIP2A, apoptosis

## Abstract

We tested the efficacy of lapatinib, a dual tyrosine kinase inhibitor which interrupts the HER2 and epidermal growth factor receptor (EGFR) pathways, in a panel of triple-negative breast cancer (TNBC) cells, and examined the drug mechanism. Lapatinib showed an anti-proliferative effect in HCC 1937, MDA-MB-468, and MDA-MB-231 cell lines. Lapatinib induced significant apoptosis and inhibited CIP2A and p-Akt in a dose and time-dependent manner in the three TNBC cell lines. Overexpression of CIP2A reduced lapatinib-induced apoptosis in MDA-MB-468 cells. In addition, lapatinib increased PP2A activity (in relation to CIP2A inhibition). Moreover, lapatinib-induced apoptosis and p-Akt downregulation was attenuated by PP2A antagonist okadaic acid. Furthermore, lapatinib indirectly decreased CIP2A transcription by disturbing the binding of Elk1 to the CIP2A promoter. Importantly, lapatinib showed anti-tumor activity in mice bearing MDA-MB-468 xenograft tumors, and suppressed CIP2A as well as p-Akt in these xenografted tumors. In summary, inhibition of CIP2A determines the effects of lapatinib-induced apoptosis in TNBC cells. In addition to being a dual tyrosine kinase inhibitor of HER2 and EGFR, lapatinib also inhibits CIP2A/PP2A/p-Akt signaling in TNBC cells.

## INTRODUCTION

Triple-negative breast cancer (TNBC), up to 15% of breast cancer, is defined as absence of estrogen receptor (ER), progesterone receptor (PR) and human epidermal growth factor receptor 2 (HER2) expression. TNBC is a subtype breast cancer with aggressive behavior, advanced disease status and poor prognosis. Because of the lack of targeting agents and limited therapeutic options, treatment of TNBC remains a great clinical challenge.

Cancerous inhibitor of protein phosphatase 2A (CIP2A) was originally identified as a cellular protein phosphatase 2A (PP2A) inhibitor, and has been shown to control oncogenic cellular signals by suppressing the tumor suppressor PP2A [1, 2]. CIP2A overexpression has been found in several human malignancies including breast cancer, hepatocellular carcinoma, gastric cancer, head and neck cancer, colon cancer, prostate cancer and non-small cell lung cancer [1-9]. In addition, CIP2A promotes the malignant growth of breast cancer cells and correlates with poor prognosis [3, 10]. Recently a comprehensive review by De *et al*. proposed an interconnected regulatory network (oncogenic nexus) of CIP2A [1]. This nexus is established through either direct interactions of CIP2A or indirectly through interactions of the CIP2A-PP2A with either multiple key cellular proteins/transcription factors or with components of key oncogenic pathways [1]. For example, through suppressing the serine/threonine phosphatase function of PP2A, CIP2A can activate oncogenic proteins such as c-Myc, ERK, and Akt [1, 11, 12]. Moreover, as an oncogenic protein in the transformation of cells and cancer progression, CIP2A has been shown to correlate with a number of drug effects in cancer [1]. In our previous studies, we demonstrated that pharmacological decrease of CIP2A, thereby increased PP2A activity and subsequent inactivation of the p-Akt signaling, inhibited proliferation and induced apoptosis in breast cancer cells [13, 14]. Furthermore, recent reviews have extensively outlined approaches to circumvent multiple challenges for CIP2A-based drugs to be established as a conventional and effective anticancer therapy in the clinics [1, 15, 16]. The comprehensive review by Khanna *et al* [15] summarized that CIP2A overexpression is found in almost all solid cancers and in some hematological malignancies such as acute and chronic myeloid leukemia, and that high expression of CIP2A has been proposed as a useful biomarker that predicts therapeutic response to chemotherapeutics such as doxorubicin, cisplatin, bortezomib, erlotinib, Checkpoint Kinase 1 inhibitors and pro-senescence based therapies such as vinka alkaloids chemotherapy and several in development small molecules [15, 17, 18]. Together, these data suggest that CIP2A plays an important role in breast cancer cells and that targeting CIP2A could be a new therapeutic option.

Lapatinib, an orally active small molecule that inhibits the tyrosine kinases of HER2 and epidermal growth factor receptor (EGFR), is approved by the US Food and Drug Administration (FDA) for patients with HER2-positive metastatic breast cancer. Furthermore, inhibition of p-ERK, p-Akt, cyclin D1 and transforming growth factor alpha, are also related in lapatinib-induced HER2-positive breast cancer cell apoptosis [19-24]. Several studies have demonstrated that lapatinib in the neoadjuvant setting achieved higher pathological complete response [25-28]. A phase III study revealed that the combination of lapatinib and capcitabine is effective in previously treated metastatic HER2-positive breast cancer [29]. Interestingly, lapatinib had an antiproliferative effect in HER2-negative breast cancer or TNBC cells [30-33]. These findings suggest that lapatinib might have certain HER2 independent anticancer properties. However, little has been explored regarding the drug effects and mechanisms of lapatinib in HER2-negative breast cancer cells. In this present study, we tested the efficacy of lapatinib in a panel of TNBC cells and examined the drug activity. We further reported the apoptotic effect and mechanism of lapatinib in TNBC cells. We found that CIP2A correlated with the effect of lapatinib in TNBC cells.

## RESULTS

### Lapatinib induced apoptosis in triple negative breast cancer cells

**Figure 1:**
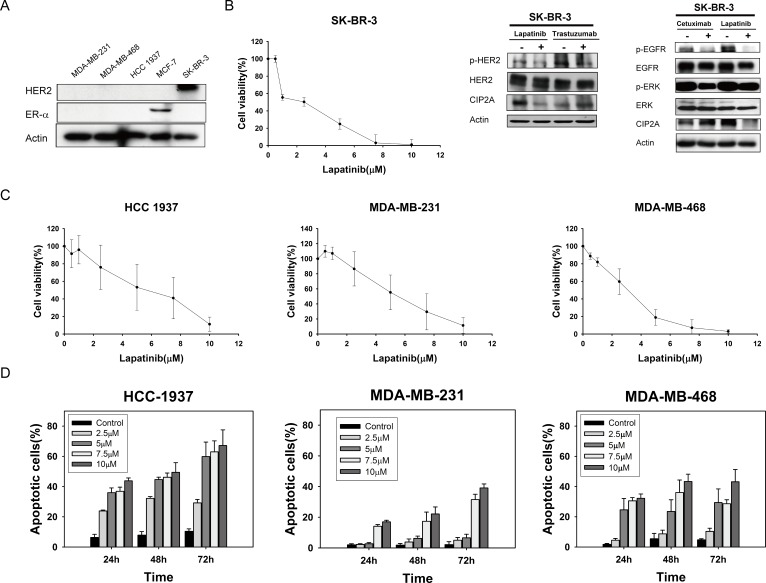
Lapatinib exerts anti-proliferative and apoptotic-inducing effects in triple-negative breast cancer (TNBC) cells **A.** Confirmation of HER2 and ER-alpha expression in TNBC cell lines (MDA-MB-231, MDA-MB-468, and HCC-1937). MCF-7 was used as a positive control for ER expression and SK-BR3 was used as a positive control for HER2 expression. **B.** Left, dose-escalation effects of lapatinib on cell viability; Middle and Right, effects of lapatinib (5 μM), anti-HER2 monoclonal antibody trastuzumab (40 μg/ml), or anti-EGFR monoclonal antibody cetuximab (20 μg/ml) on CIP2A in HER2-positive SK-BR3 cells. HER2, p-HER2, EGFR, p-EGFR, ERK, and p-ERK were assayed to confirm the target effects of these drugs. **C.** Dose-escalation effects of lapatinib on cell viability in TNBC cells. Cells were exposed to lapatinib at the indicated doses for 72 hours and cell viability was assessed by MTT assay. *Points*, mean (*n* = 3); *bars*, SD. **D.** Dose-and time-escalation effects of lapatinib on apoptosis in TNBC cell lines. Cells were exposed to lapatinib at the indicated doses for 24, 48, and 72 hours. Apoptotic cells were determined by flow cytometry (sub-G1 analysis of *propidium iodide*-stained cells). *Columns*, mean (*n* = 3); *bars*, SD.

To investigate the apoptosis effect induced by lapatinib, we tested three TNBC cell lines: MDA-MB-231, MDA-MB-468, and HCC-1937. The triple negative characteristics of all cell lines were substantiated by western blotting. MCF-7 was used as a positive control for ER expression and SK-BR3, an HER2 positive breast cancer cell line, was a positive control for HER2 expression (Figure 1A). Since lapatinib is a dual EGFR/HER2 kinase inhibitor, we first examined the target effects (on HER2 and EGFR signals) of lapatinib in HER2-positive SK-BR3 cells. As shown in Figure 1B, MTT test confirmed the antiproliferative effect of lapatinib on SK-BR-3. Lapatinib, and trastuzumab, an anti-HER2 monoclonal antibody, both revealed inhibition of p-HER2 in SK-BR3. Similarly, lapatinib and cetuximab, an anti-EGFR monoclonal antibody, both downregulated p-EGFR and p-ERK in SK-BR3. Interestingly, only lapatinib demonstrated CIP2A inhibition, and both anti-EGFR or anti-HER2 monoclonal antibodies had no effects on CIP2A (Figure 1B, right). Furthermore, lapatinib elicited apoptosis in MDA-MB-231, MDA-MB-468, and HCC-1937 cells in a dose-dependent manner (Figure 1C). Flow cytometric detection of sub-G1 cells at the indicated times (24, 48 and 72 h) and doses (2.5, 5, 7.5 and 10 μM) also demonstrated that lapatinib induced apoptosis (Figure 1D). To summarize, lapatinib-induced apoptosis in MDA-MB-231, MDA-MB-468, and HCC-1937 cells is both dose- and time-dependent. These results indicated that TNBC cell lines MDA-MB-231, MDA-MB-468, and HCC-1937, as well as HER2 positive cell line SK-BR-3, are sensitive to the cytotoxic effect of lapatinib.

### Lapatinib induces apoptosis through inhibition of CIP2A and p-Akt in sensitive triple negative breast cancer cells

**Figure 2:**
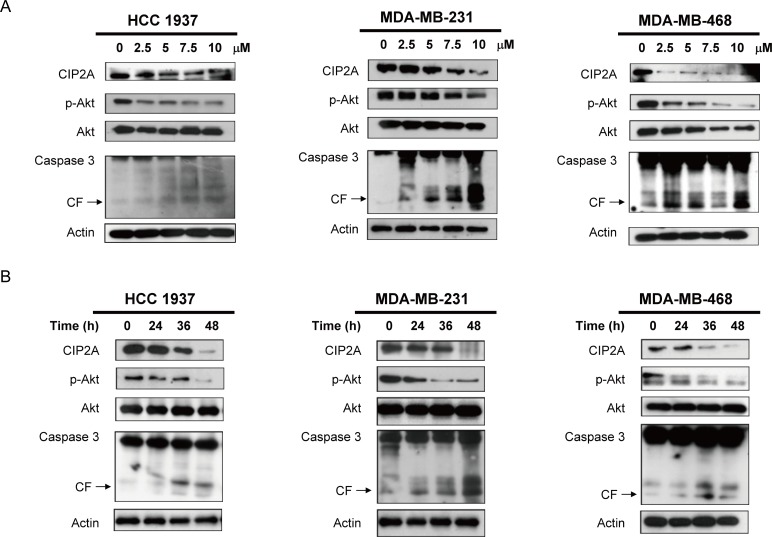
Lapatinib induces apoptosis in association with downregulation of CIP2A and p-Akt in TNBC cells **A.** Dose-escalation effects of lapatinib on CIP2A, p-Akt, and caspase 3 cleavage. Cells were exposed to lapatinib at the indicated doses for 48 hours. **B.** time-dependent analysis of CIP2A, p-Akt, and caspase 3 cleavage. Cells were exposed to lapatinib (10 μM) for 24, 36 and 48 hours. Cell lysates were prepared and assayed for these molecules by western blotting. Data are representative of three independent experiments. Apoptotic cells were determined by flow cytometry (sub-G1 analysis of *propidium iodide*-stained cells).

In our previous study, we found that bortezomib induced apoptosis in TNBC cells through downregulation of CIP2A and p-Akt, suggesting that CIP2A is a target of bortezomib [13]. Therefore, in this study, we hypothesized that CIP2A may also play a role in the molecular events associated with apoptosis induced by lapatinib in TNBC cells. As demonstrated in Figure 2A, the protein level of CIP2A and p-Akt decreased significantly in proportion to the lapatinib concentration in MDA-MB-231, MDA-MB-468, and HCC-1937 cells after treatment for 48 hours. Lower CIP2A and p-Akt levels were also noted when cells were treated with lapatinib (10 μM) for other treatment periods (24, 36, 48 h) (Figure 2B).

### Validation of the CIP2A/p-Akt pathway as a molecular determinant of lapatinib induced TNBC apoptosis

**Figure 3:**
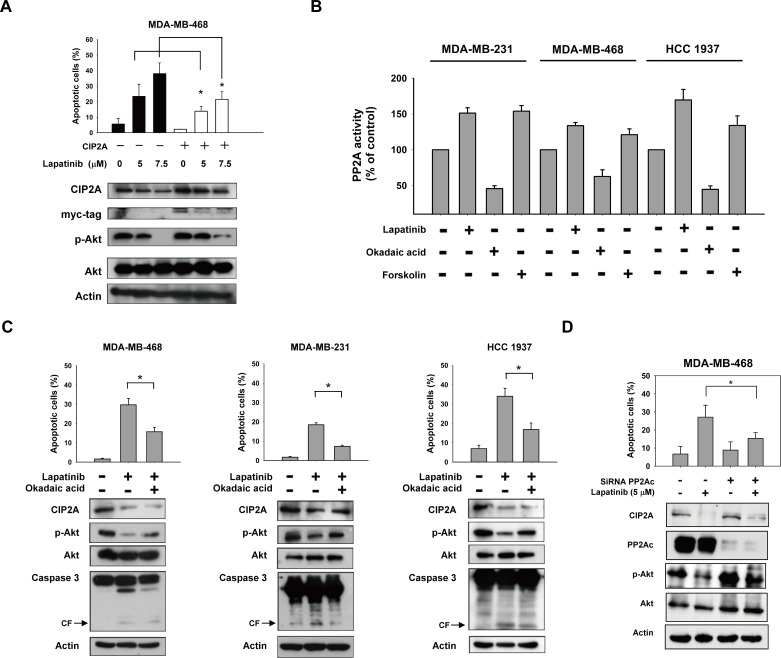
CIP2A/PP2A/p-Akt mediates lapatinib-induced apoptosis in TNBC cells **A.** ectopic expression of myc-tagged CIP2A reduced the apoptotic effect of lapatinib in MDA-MB-468 cells. **B.** Analysis of PP2A activity in drug-treated TNBC cells. Cells were treated with DMSO or lapatinib at 10 μM or okadaic acid at 20 nM (as a negative control) or forskolin at 40 μM (as a positive control) for 24 hours. Cell lysates were assayed for PP2A activity. **C.** Pretreatment of PP2A inhibitor okadaic acid protected cells from lapatinib-induced apoptosis. Cells were pretreated with okadaic acid (20 nM) for 1 hour; then washed and treated with DMSO (control) or lapatinib (10 μM) for 24 hours. Cell lysates were separated and assayed for sub-G1 analysis and western blotting. **D.** Knockdown of PP2Ac reduced the effects of lapatinib on p-Akt and apoptosis. MDA-MB-468 cells were transfected with siRNA against PP2Ac (catalytic subunit) or control siRNA for 48h, after transfection cells were then treated with lapatinib 5μM for 24 h. Cell lysates were separated and assayed for sub-G1 analysis and western blotting.

To examine the role of CIP2A and p-Akt in mediating lapatinib-induced apoptosis, we generated MDA-MB-468 cells with constitutive, ectopic expression of myc-tagged CIP2A. Lapatinib-induced apoptosis was reduced significantly in CIP2A overexpressing MDA-MB-468 cells (Figure 3A). Because CIP2A is a cellular inhibitor of PP2A [2, 34], we tested the PP2A activity in lapatinib-treated cells. As shown in Figure 3B, lapatinib significantly increased the phosphatase activity of PP2A in lapatinib-sensitive cell lines. In addition, okadaic acid, a PP2A inhibitor acting as a negative control, decreased the phosphatase activity of PP2A in these three cell lines; while forskolin, a PP2A agonist acting as a positive control, increased PP2A activity in the cells (Figure 3B). Moreover, pretreatment with okadaic acid eliminated the effects of lapatinib on apoptosis in lapatinib-sensitive MDA-MB-231, MDA-MB-468 and MDA-MB-453 cells (Figure 3C). Next we performed siRNA of PP2Ac (Catalytic subunit of PP2A enzyme complex) and examine the role of PP2A in lapatinib-induced effects. As shown in Figure 3D, since CIP2A is an endogenous inhibitor of PP2A, knockdown PP2Ac did not significantly alter CIP2A protein expression or lapatinib-induced CIP2A inhibition (row 1, Figure 3D western blot). However, knockdown PP2Ac by siRNA reduced lapatinib-induced apoptosis (Figure 3D, upper panel), in association with increased PP2A phosphatase-related p-Akt expression (row 3, Figure 3D western blot). The result suggested knockdown of PP2Ac partially reversed the effects of lapatinib on p-Akt and apoptosis, supporting the role of PP2A in lapatinib-induced apoptosis and p-Akt inhibition. Together, these results suggest that lapatinib mediates apoptosis in TNBC cells through the CIP2A/PP2A/p-Akt pathway.

### Lapatinib inhibits transcription of CIP2A in TNBC cells

**Figure 4:**
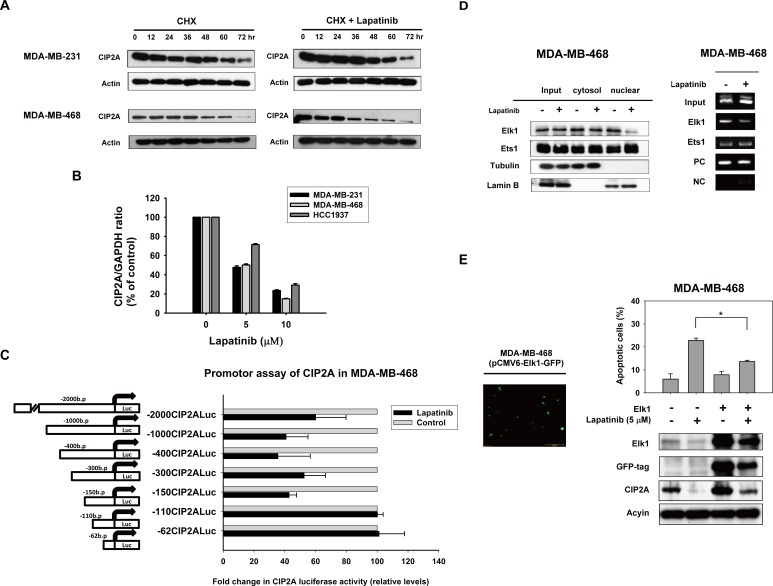
Lapatinib inhibits transcription of CIP2A **A.** Effect of lapatinib on CIP2A protein degradation. Cells were treated with 100 μg/ml pan-translation inhibitor cyclohexamide (CHX) in the presence (right) or absence (left) of lapatinib (5 μM) for the indicated period of time, then the stability of CIP2A protein in whole-cell lysates was assessed by western blot. The addition of lapatinib did not significantly affect CIP2A degradation. **B.** Lapatinib inhibited CIP2A transcription. Cells were treated with lapatinib at the indicated doses for 24 hours, after which total RNA was isolated and CIP2A mRNA was assayed by real-time quantitative PCR. *Columns*, mean (*n* = 3); *bars*, SD. **C.** Luciferase reporter assay of *CIP2A* proximal promoter regions upon lapatinib treatment. MDA-MB-468 cells were transfected by Firefly luciferase reporter vectors carrying *CIP2A* promoters of different lengths as indicated, and Renilla vectors for 24 hours and then treated with 5 μM lapatinib or DMSO for 24 hours. Cell lysates were then assayed for dual luciferase activity as described in Materials and Methods. *Columns*, mean (*n* = 3); *bars*, SD; *, *P* < 0.05. **D.** lapatinib disturbed binding of Elk1 to the CIP2A promoter region. Left, lapatinib decreased Elk1 translocation from the cytosol to the nuclei. Nuclear and cytoplasmic extracts were prepared from MDA-MB-468 cells treated with lapatinib (5 μM) or DMSO for 24 hours. Cell lysates were western blotted for Elk1, and Ets1. Lamin B and Tubulin were used as a loading control. Right, chromatin immunoprecipitation assays of the CIP2A promoter were performed as described in Materials and Methods. Soluble chromatin was immunoprecipitated with Elk1, Ets1 or IgG (negative control) antibodies. Immunoprecipitates were subjected to PCR with primer pairs specific to the CIP2A promoter (−16 to −139 bp). The gel shown is representative of three independent experiments. Anti-RNA polymerase II antibody and GAPDH primers were used as a positive control for the assay technique and reagent integrity. **E.** Ectopic expression of Elk1 with pCMV-Elk1-GFP vector upregulated CIP2A expression, and suppressed lapatinib-induced CIP2A inhibition.

To further study how lapatinib inhibited CIP2A expression, we first tested whether lapatinib could increase CIP2A elimination (degradation) when translation was blocked by the protein synthesis inhibitor cycloheximide (CHX). Our data showed that after protein translation was blocked by CHX, the rate of CIP2A degradation did not change significantly with or without lapatinib treatment in MDA-MB-231 and MDA-MB-468 cells (Figure 4A). This suggested that the effect of lapatinib on CIP2A may occur at the pre-translation level. We next investigated whether lapatinib suppressed CIP2A transcription via real-time quantitative PCR analysis. As shown in Figure 4B, CIP2A mRNA levels decreased in a dose-dependent manner upon treatment with lapatinib in sensitive MDA-MB-231 and MDA-MB-468, HCC-1937 cells (Figure 4B). Therefore, we hypothesized that lapatinib inhibited CIP2A at the transcription level.

To further illustrate the possible mechanism through which lapatinib reduced CIP2A mRNA, we assumed that lapatinib may inhibit CIP2A promoter activity through transcription factor(s), since some studies have unraveled several transcriptional regulators of the CIP2A promoter [35, 36]. Accordingly, MDA-MB-468 cells were transfected with luciferase reporter constructs for CIP2A promoter of varying lengths. As shown in Figure 4C, lapatinib significantly downregulated the activity of CIP2A promoter in cells transfected with constructs of −1 to −2000, −1 to −1000, −1 to −400, −1 to −300 and −1 to −150, respectively, while this promoter activity reduction was not found when cells were transfected with constructs of −1 to −62 and −1 to −110 bp. According to previous studies [35, 36], Ets1 and Elk1 could bind to promoter regions between −400 to −110 bp. Therefore, we performed chromatin immunoprecipitation assay (ChIP) assay to examine whether the binding of Ets1 or Elk1 (or both) to CIP2A promoter was inhibited by lapatinib. We found that lapatinib disturbed the binding of Elk1 to CIP2A promoter. Further western blotting of Elk1 in nuclear/cytoplasmic extracts from MDA-MB-468 cells treated with or without lapatinib revealed that lapatinib reduced Elk-1 expression in the nuclear extracts (Figure 4D). These data suggest that lapatinib may decrease CIP2A transcription via inhibiting the binding of Elk1 to the CIP2A promoter. We also validate the role of Elk1 in lapatinib-induced CIP2A inhibition by ectopic expression of Elk1 with pCMV-Elk1-GFP vector. As shown in Figure 4E, ectopic Elk1 expression upregulated CIP2A expression, and suppressed lapatinib-induced CIP2A inhibition.

### Effect of lapatinib on triple negative breast cancer xenograft tumor growth *in vivo*

**Figure 5:**
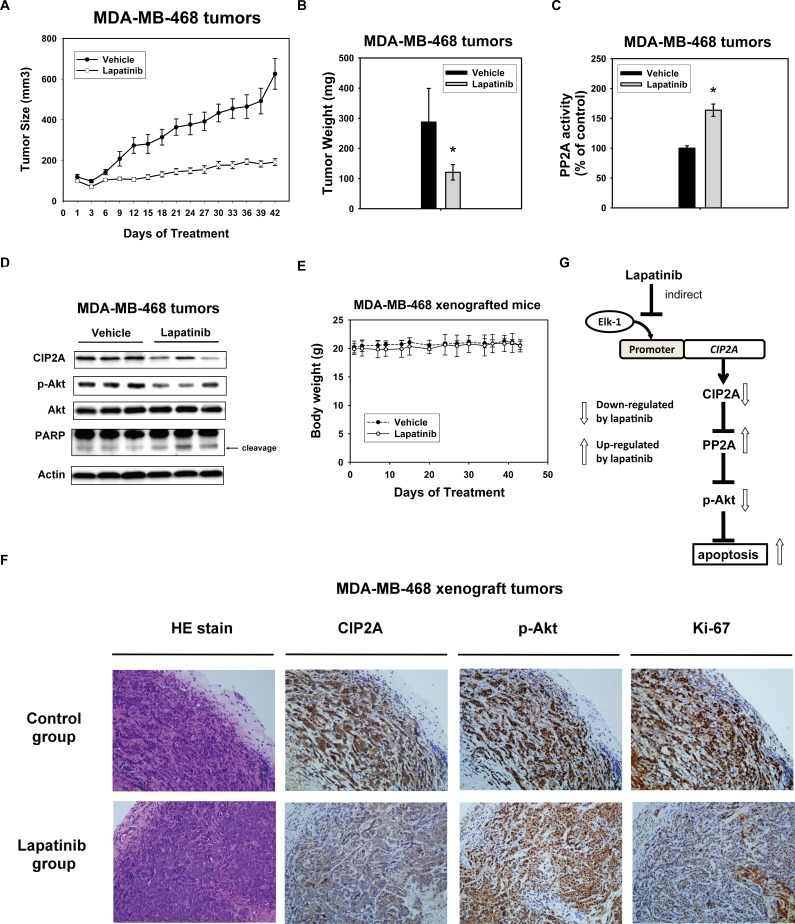
*In vivo* effect of lapatinib on MDA-MB-468 xenograft nude mice **A.** Lapatinib treatment decreased the size of MDA-MB-468 tumors. *Points*, mean (*n* = 6); *bar*, SE. *, *P* < 0.05. Mice were administered orally with 100 mg/kg body weight lapatinib three times weekly or vehicle (1× phosphate buffered saline), as described in Materials and Methods. **B.** tumor weight and **C.** PP2A activity in MDA-MB-468 xenografts treated with control or lapatinib. *Columns*, mean (*n* = 3); *bars*, SD; *, *P* < 0.05. **D.** Western blot analysis of the expression level of CIP2A, pAkt and Akt in MDA-MB-468 xenografts treated with control or lapatinib. **E.** Body weights of xenograft mice bearing tumors. *Points,* mean (*n* = 6); *bar*, SD. **F.** Hematoxylin and eosin (HE) stain, and immunohistochemical (IHC) staining for CIP2A, p-Akt and the proliferative index Ki-67 in the MDA-MB-468 xenografts treated with control or lapatinib. Power fields 200X. **G.** schema of the molecular mechanism of the action of lapatinib on the CIP2A/PP2A pathway. By indirectly inhibiting CIP2A transcription, lapatinib restores PP2A activity downregulating p-Akt and leading to subsequent cell apoptosis.

In order to confirm that using lapatinib to inhibit CIP2A is potentially clinical relevant in TNBC, we next used TNBC xenograft models to evaluate the effect of lapatinib *in vivo*. MDA-MB-468 xenograft was generated to validate the role of CIP2A *in vivo*. After successfully establishing the xenograft model in nude mice, these tumor-bearing mice were treated with lapatinib at a dose of 100 mg/kg or vehicle (as control) orally three times weekly for 7 weeks. As shown in Figure 5A and 5B, lapatinib inhibited MDA-MB-468 tumor size and tumor weight significantly. PP2A activity in MDA-MB-468 xenografts treated with lapatinib increased significantly compared with the control group (Figure 5C). Furthermore, the protein expression of CIP2A, p-Akt and Akt were checked to confirm the correlation between the biological response observed *in vivo* and the molecular mechanism discovered *in vitro*. Lapatinib increased the expression of CIP2A and p-Akt consistently in the three representative MDA-MB-468 tumors, while no change was seen in the control (vehicle)-treated tumors (Figure 5D). At the end of the experiment, all the animals had tolerated the treatments quite well without observable signs of toxicity and had stable body weights throughout the whole treatment course (Figure 5E). Moreover, we performed hematoxylin and eosin (HE) stain, and immunohistochemical (IHC) staining for CIP2A, p-Akt and the proliferative index Ki-67 in the MDA-MB-468 xenografts. As shown in Figure 5F, xenograft tumors from lapatinib treatment group showed reduced expressions of CIP2A, p-Akt, and ki-67 in the cancer cells population, defined by HE stain (Figure 5F). A schema summarizing the molecular mechanism of lapatinib in sensitive TNBC cells is presented in Figure 5G. Lapatinib indirectly inhibited CIP2A transcription by disturbing the binding of Elk1 to the CIP2A promoter and restoring PP2A activity, which led to p-Akt downregulation and cancer cell apoptosis.

## DISCUSSION

In this study, we demonstrated a novel mechanism through which lapatinib induces apoptosis in TNBC cells, i.e., through ELK-1-related CIP2A downregulation. We further identified CIP2A as a major molecular determinant of the apoptosis-inducing effect of lapatinib in TNBC cells. First, we found that downregulation of CIP2A and p-Akt correlated with lapatinib-induced apoptosis in TNBC cells. Moreover, overexpression of CIP2A upregulated p-AKT and protected sensitive MDA-MB-468 cells from lapatinib-induced apoptosis. Second, lapatinib inhibited xenograft tumor growth *in vivo* in association with CIP2A downregulation. Third, lapatinib indirectly downregulated CIP2A transcription by disturbing the binding of Elk1 to the CIP2A promoter. These findings not only increase current understanding of the drug mechanisms of lapatinib but also support the rationale for targeting CIP2A in future development of therapies for TNBC.

Our findings strengthen the evidence for the use of CIP2A as an anti-cancer target. Several studies support the use of CIP2A as an anticancer target [2-4, 10, 34, 37-42]. Accordingly, several agents that inhibit CIP2A have been identified, and some have demonstrated efficacy against different cancer cells. In our previous studies, we demonstrated that bortezomib, a proteasome inhibitor, induced apoptosis via proteasome-independent inhibition of CIP2A in TNBC cells [13], hepatocellular carcinoma cells [41], leukemia cells [34] and head and neck cancer cells [40]. In this study, we found that lapatinib, a dual tyrosine kinase inhibitor of HER2 and EGFR, induced significant cancer cell apoptosis in TNBC (Figure 1). We confirmed the role of the CIP2A/PP2A/p-Akt signaling pathway in lapatinib-induced apoptosis in TNBC cells. It is noteworthy the concentrations of lapatinib used in current study were several fold higher than previous studies [31, 32], we also tested whether lapatinib had effects in TNBC cells at lower concentrations. As shown in Supplement Figure S1, lower concentrations of lapatinib (0.1 and 1 μM) had little effects on TNBC cells in terms of apoptosis and CIP2A protein expression. This result suggested that higher doses of lapatinib are required for lapatinib-induced CIP2A inhibition and apoptosis in TNBC cells.

Prior studies have indicated the role of CIP2A in senescence induction, that CIP2A may render breast cancer cells resistance to senescence induction [16, 43]. Moreover, Come *et al* [3] has demonstrated that CIP2A depletion by siRNA resulted in a decrease of the anchorage-independent growth of MDA-MB-231 cell. Li et al [38] also showed CIP2A depletion by siRNA lead to impaired clonogenicity and senescence in gastric cancer cells. It would be interesting to see whether lapatinib-induced CIP2A downregulation is also associated with clonogenicity and senescence. Accordingly, we performed colony-forming assay and senescence-associated beta-galactosidase (SA-β-gal) assay. As shown in Supplement Figure S2, lapatinib at dose of 5 μM, but not at lower doses (0.1 and 1 μM), significantly suppressed the colony forming ability of MDA-MB-231 cells. The result was in consistence with that by Chen *et al* [31], which also showed that lapatinib at 1 μM did not suppress colony forming ability of MDA-MB-231 cells (in their Figure 6B). In contrast, lapatinib induced only minor cell senescent-phenotype in MDA-MB-231 cells, and not in a dose-dependent manner. Previously, Angelini *et al* [44] showed that lapatinib could not revert the HER2-induced senescence phenotype in a constitutive active HER2 (+) (MCF7 Tet-Off/p95HER2) cells, whereas McDermott *et al* [45] showed lapatinib induced senescent-like phenotype in HER2 positive HCC1419 breast cancer cells. Together with these findings and that lapatinib decreased CIP2A at doses of 5 μM and higher, we suggested that lapatinib-induced CIP2A inhibition may also suppress clonogenicity in TNBC cells. It remains inconclusive the role of lapatinib-induced CIP2A inhibition in senescence induction and more studies are necessary.

Elk-1, a member of the ETS family of transcription factors, is involved in a number of important cellular processes in a number of normal tissues as well as in many malignancies [46]. In particular, in cancer cells Elk-1 has been shown to correlate with tumor progression via activation of different genes that regulate cell growth, differentiation, and survival [47, 48]. The role of Elk-1 in cancer cell formation has been progressively revealed over the past few years [49-52]. Furthermore, Elk-1 has been found to physiologically regulate CIP2A. Pallai *et al.* demonstrated that Elk-1 and Ets-1 are both required to coordinate binding to the proximal CIP2A promoter for CIP2A expression in cervical, endometrial and liver carcinoma cell lines [36]. In contrast, Khanna *et al.* showed that only Ets-1 is required for the regulation of CIP2A expression in prostate and gastric carcinoma [35]. It has been suggested that different transcription factors regulate CIP2A expression in a cell-type specific manner. According to the Figure 4C and 4D results, lapatinib may disturb Elk1 binding to CIP2A promoter in TNBC cells. Since Elk1 can also regulate Mcl-1 transcription, an anti-apoptotic protein, by binding to Mcl-1 promoter [53]. We used primers of Mcl-1 promoter as a positive control for ChIP assay. As shown in Supplement Figure S3, ChIP assay demonstrated that immunoprecipitated Elk1 protein bound with Mcl-1 and CIP2A promoter DNA fragments. Interestingly, lapatinib did not significantly inhibit Elk1 binding to Mcl-1, suggesting lapatinib disturbed Elk1-CIP2A but not Elk1-Mcl-1 protein-DNA interaction, more studies are necessary to further dissect the mechanism. However, non-specificity issues may exist at relatively higher doses of lapatinib used in luciferase promoter assays. We used luciferase reporter constructs for Mcl-1 promoter (pGL2-Mcl-1) as a positive control for luciferase promoter assays. As shown in Supplement Figure S4, treatment with lapatinib at 5 μM suppressed about one-fourth the luciferase reporter activity. As a specific kinase inhibitor of EGFR and HER2, lapatinib has been demonstrated to affect multiple cellular pathways downstream of EGFR and HER2 inhibition [54, 55]. Moreover, Mcl-1 expression can be enhanced by EGFR-dependent transcriptional regulation [56]. Therefore, it is possible that lapatinib decreased Mcl-1 promoter activity through EGFR-dependent signaling or yet-to-be identified transcriptional signals other than Elk1-Mcl-1. Nevertheless, we have validated the role of Elk-1 in lapatinib-induced CIP2A downregulation; ectopic Elk1 expression upregulated CIP2A expression, and suppressed lapatinib-induced CIP2A downregulation (Figure 4E). Based on aforementioned studies and our results, we suggest that any attempt to therapeutically decrease CIP2A expression through inhibition of specific transcription factors will need to be guided by the type of cancer that is being treated. These studies also suggested that CIP2A regulation is quite complex and needs further investigation to be thoroughly understood.

Because lapatinib is an EGFR/HER2 kinase inhibitor, it would be interesting to see whether other similar kinase inhibitors may also inhibit CIP2A expression. We further tested neratinib (formally HKI-272) and afatinib, both of which are pan epidermal growth factor receptor (ErbB) family inhibitor [57], to demonstrate their effects on p-EGFR, p-HER2 and on CIP2A. As shown in supplement Figure S5, both neratinib and afatinib decreased p-EGFR and p-HER2 but did not inhibit CIP2A expression in SK-BR3 cells. In comparison, lapatinib decreased CIP2A, p-EGFR, and p-HER2. All three tyrosine kinase inhibitors induced apoptosis, as evident by PARP cleavage. In line with the data shown in Figure 1B that both anti-EGFR or anti-HER2 monoclonal antibodies had no effects on CIP2A, our results suggested lapatinib-induced CIP2A inhibition may not be necessarily associated with tyrosine-kinase inhibition of EGFR and HER2. However, more studies are needed to fully address the correlation.

Although most clinical evidence suggests that lapatinib is particularly effective for HER2-positive breast cancer because it acts by reversible inhibition of the HER2 receptor [19, 20, 22, 23], the antitumor effect of lapatinib in HER2-negative breast cancer continues to be of interest. EGFR expression has been reported to exist in 45% to 76% of cases of TNBC [58-61]. The selective EGFR-targeting and clinical benefit of lapatinib in cancer progression and metastasis indicate that lapatinib might be a candidate therapeutic drug for TNBC. Unfortunately, the antiproliferative effect of lapatinib on TNBC cells *in vitro* did not transfer into solid efficacy *in vivo*. Chen *et al* [31] showed that lapatinib (at dose of 1 μM) could induce nuclear factor (NF)-κB activation, independent of EGFR/HER2 inhibition, in TNBCs. They found that lapatinib, but not other EGFR inhibitors synergized the anti-tumor activity of proteasome inhibitors both *in vitro* and *in vivo*, suggesting that treatment of TNBCs with lapatinib may enhance their oncogene addiction to NF-κB, and thus augment the anti-tumor activity of proteasome inhibitors. On the other hand, Hsia *et al* found an opposing effect of lapatinib in TNBC that lapatinib may increase the migration and invasion of MDA-MB-231 cells by upregulating EGFR and COX-2 through the downregulation of microRNA-7, providing a potential explanation for the worse clinical outcome of TNBC patients who receive lapatinib-based treatment [62]. It remains unknown whether and how lapatinib elicits the aggressiveness of such cancer cells. Meanwhile, it would be interesting to see cells that may be resistant to lapatinib-induced CIP2A inhibition and discover the resistance mechanism in the future.

The current study also has some limitation. First, despite we showed Elk1 plays a role in lapatinib-induced CIP2A inhibition (Figure 4D to 4E), the detail mechanism by which lapatinib suppresses the CIP2A promoter remains unsolved thereby represents a limitation of current study. Future experiments using a site-directed mutagenesis of the specific cellular component that is affected or brought into action following the treatment of lapatinib may help delineate the mechanism. Secondly, whereas we showed siRNA PP2Ac treatment reduced lapatinib-induced decrease of p-Akt (Figure 3D), the lowering of p-Akt can also be contributed by lapatinib's kinase-inhibitory effects on the EGFR/HER2 signaling pathways (independent of CIP2A-PP2A axis). Moreover, although various studies have indicated the clinical relevance and prognostic value of CIP2A in various cancers [1, 15], its implication as a therapeutic target for TNBC remains to be validated. We also performed *in silico* analysis of CIP2A mRNA expression (mRNA seq v2) from The Cancer Genome Atlas (TCGA) public data portal (https://tcga-data.nci.nih.gov/tcga/). As shown in Supplement Figure S6, Elk1 and CIP2A showed higher mRNA expression in tumor versus adjacent normal tissue from all breast cancer patient samples, from paired tumor/normal tissue and from the TNBC subpopulation. Future more clinical studies are required to identify CIP2A upregulation or overexpression specifically in patients with TNBC and correlate the expression or downstream function of the protein with the clinical outcome in these patients.

## CONCLUSIONS

Lapatinib shows a favorable apoptosis-inducing effect in TNBC cells through a novel mechanism, i.e., the Elk-1/CIP2A/PP2A/p-Akt pathway. This study strengthens the notion that CIP2A is a major molecular determinant of the sensitivity of TNBC cells to lapatinib-induced apoptosis. Targeting the interactions of transcription factors, oncoproteins, phosphatases and kinases could be a novel anti-cancer strategy. Future studies to explicate the mechanism by which lapatinib inhibits Elk-1 may lead to further progress in the development of molecular-targeted therapies for TNBC.

## MATERIALS AND METHODS

### Reagents and antibodies

Lapatinib, okadaic acid and forskolin for *in vitro* experiments were purchased from Cayman Chemical (Ann Arbor, MI, USA). Lapatinib ditosylate (Tykerb) tablets obtained from GlaxoSmithKline (London, UK) were used for *in vivo* animal experiments. For *in vitro* studies, lapatinib at various concentrations was dissolved in dimethyl sulfoxide (DMSO) and added to cells in Dulbecco's modified Eagle's medium (Invitrogen, Carlsbad, CA, USA). The final DMSO concentration was 0.1% after addition to the medium. Antibodies for immunoblotting of CIP2A and ER-α, Ets1, and Elk1 were obtained from Santa Cruz Biotechnology (San Diego, CA, USA). Other antibodies such as Akt, p-Akt (Ser473), caspase 3 and Myc-tag were obtained from Cell Signaling (Danvers, MA, USA).

### Cell culture and western blot analysis

The HCC-1937, MDA-MB-231, MDA-MB-468, SK-BR-3 and MCF-7 cell lines were obtained from American Type Culture Collection (Manassas, VA, USA). All breast cancer cells were maintained in Dulbecco's modified Eagle's medium supplemented with 10% FBS, 0.1 mM nonessential amino acids (NEAA), 2 mM L-glutamine, 100 units/mL penicillin G, 100 μg/mL streptomycin sulfate, and 25 μg/mL amphotericin B in a 37°C humidified incubator and an atmosphere of 5% CO_2_ in air. Lysates of breast cancer cells treated with drugs at the indicated concentrations for various periods of time were prepared for immunoblotting of p-Akt, Akt, CIP2A, etc. Western blot analysis was performed as previously reported [13].

### Apoptosis analysis

Drug-induced apoptotic cell death was assessed using measurement of apoptotic cells by flow cytometry (sub-G1 analysis of *propidium iodide*-stained cells) and western blot analysis of caspase 3 cleavage.

### Generation of MDA-MB-468 cells with ectopically expressed CIP2A

CIP2A cDNA (gene name KIAA1524) was purchased from Origene (RC219918; Rockville, MD, USA) and constructed into pCMV6 vector. MDA-MB-468 cells were transfected with myc-tagged CIP2A as previously described [30]. Briefly, following transfection, cells were incubated in the presence of Geneticin (G418, 1.40 mg/mL, Invitrogen). After 8 weeks of selection, surviving colonies, i.e., those arising from stably transfected cells were selected and individually amplified.

### PP2A activity assay

The phosphatase activity of PP2A was detected by a commercial PP2A immunoprecipitation phosphatase assay kit (Millipore, Billerica, MA, USA) according to the manufacturer's instructions and as previously described [30]. In brief, drug-treated or control cells were lysed and PP2A was immunoprecipitated with anti-PP2A C subunit antibodies and protein A agarose beads overnight. Protein phosphatase activity of PP2A was determined by measuring the generation of free phosphate from threonine phosphopeptide using the malachite green-phosphate complex assay. To avoid variability due to differences in the amounts of immunoprecipitated protein between samples, the phosphatase activities were normalized to the amount of PP2A immunoprecipitated, as detected and quantified by immunoblot analysis for each treatment group.

### Luciferase reporter constructs for the CIP2A promoter and 5′ detection analysis

The upstream region of the CIP2A promoter containing exon 1 (−2000 bp to −1 bp) was amplified by PCR from the genomic DNA of cells and cloned into the reporter vector, Firefly vector (pGL4.17, Promega, Madison, WI) by KpnI and Bgl II restriction sites. PCR amplified promoter regions -2000/-1, -400/-1, -110/-1, -62/-1, were cloned into the KpnI and Bgl II restriction sites of the pGL4-basic vector. The nucleotide sequence of the clones was verified by sequencing.

### Dual-luciferase reporter assay

The promoter activity of *CIP2A* was determined using the dual-luciferase reporter assay kit (*Promega*, Madison, WI) according to the manufacturer's instructions. MDA-MB-468 cells were co-transfected with the luciferase reporter constructs pGL4.17-*CIP2A*-promoter (Firefly fluorescence reporter) and PGL4.74-renilla (Renilla fluorescence reporter) as an indicator of normalization of transfection efficiency. Twenty-four hours post-transfection, cells were treated with lapatinib (5 μM) or DMSO for 24 hours. Cells were then lysed and assayed for luciferase activity. The promoter activity was repeated three times in parallel for statistical analysis.

### Chromatin immunoprecipitation assay

Chromatin immunoprecipitation was performed using Pierce Agarose ChIP Kit (Thermo Fisher Scientific, Rockford, IL, USA) according to the manufacturer's instructions. Briefly, 1 × 10^7^ MDA-MB-468 cells were treated with lapatinib (5 μM) or DMSO for 24 hours. Physical cross-linking between chromatin (DNA) and proteins was fixed by 1% formaldehyde at room temperature for 10 min. Then, cells were lysed for DNA digestion by enzyme (Micrococcal Nuclease, 37°C, 15 min); phosphatase inhibitor and protease inhibitor were added in the cell lysis step to avoid protein degradation. Lysates were clarified by centrifugation at 12,500 × *g* for 5 min at 4°C. Immunoprecipitation was performed by adding Elk1, Ets1 or rabbit IgG antibodies as the negative control. The immunocomplex was precipitated by incubation with 25 μl of protein A/G magnetic beads for 1 h at 4°C. The protein/DNA complex was eluted using 200 μl of elution buffer from the beads. Cross-linking of protein-DNA was reversed by adding 8 μl of 5 M NaCl at 95°C for 15 minutes. The DNA was purified using spin columns and 2 μl of the DNA was used in the semi-PCR reaction for amplification of the CIP2A promoter region (−139/−16 bp). Anti-RNA polymerase II antibody and GAPDH primers were provided by the manufacturer as a positive control for assay technique and reagent integrity.

### Xenograft tumor growth

Female NCr athymic nude mice (5-7 weeks of age) were obtained from the National Laboratory Animal Center (Taipei, Taiwan, ROC). The mice were housed in groups and maintained in an SPF-environment. All experimental procedures using these mice were performed in accordance with protocols approved by the Institutional Animal Care and Use Committee of Taipei Veterans General Hospital. Each mouse was inoculated orthotopically to the mice mammary pads with 5 × 10^6^ breast cancer cells suspended in 0.1 mL serum-free medium containing 50% Matrigel (BD Biosciences, Bedford, MA) under isoflurane anesthesia. Tumors were measured using calipers and their volumes calculated using a standard formula: width^2^ × length × 0.52. When tumors reached around 200 mm^3^, mice were administered orally with 100 mg/kg body weight lapatinib three times weekly for 7 weeks. Controls received vehicle (1× phosphate buffered saline). Upon termination of treatment, mice were sacrificed and xenografted tumors were harvested and assayed for tumor weight, PP2A activity, and molecular events by western blot analysis.

### Reverse transcription-polymerase chain reaction

Total RNA was extracted from cultured cells using TRIzol Reagent (Invitrogen, San Diego, CA) and RT-PCR was performed according to the manufacturer's instructions (MBI Fermentas, Vilnius, Lithuania). RT-PCR analyses were performed as previously described [13], using specific primers for human CIP2A (forward primer, 5′-TGGCAAGATTGACCTGGGATTTGGA-3′; reverse primer, 5′-AGGAGTAATCAAACGTGGGTCCTGA-3′; 172 bps), and the glyceraldehyde-3-phosphate dehydrogenase (GAPDH) gene was chosen as an internal control (forward primer, 5′-CGACCACTTTGTCAAGCTCA-3′; reverse primer, 5′-AGGGGTCTACAT GGCAACTG-3′; 228 bps). Real-time quantitative PCR was performed in a LightCycler 480II instrument (Roche Diagnostics, Indianapolis, IN, USA) using a LightCycler 480 SYBR Green I Master kit (Roche Diagnostics). Primers are the same as described above.

### Statistical analysis

Data are expressed as mean ± SD or SE. Statistical comparisons were based on nonparametric tests and statistical significance was defined as P value less than 0.05. For survival analysis, progression-free survival curves of patients were generated by the Kaplan-Meier method and compared by log rank test. All statistical analyses were performed using SPSS for Windows software, version 12.0 (SPSS, Chicago, IL, USA).

## SUPPLEMENTARY MATERIAL AND METHODS FIGURES


