# Correction: Lapatinib inhibits CIP2A/PP2A/p-Akt signaling and induces apoptosis in triple negative breast cancer cells

**DOI:** 10.18632/oncotarget.15112

**Published:** 2017-02-06

**Authors:** Chun-Yu Liu, Ming-Hung Hu, Chia-Jung Hsu, Chun-Teng Huang, Duen-Shian Wang, Wen-Chun Tsai, Yi-Ting Chen, Chia-Han Lee, Pei-Yi Chu, Chia-Chi Hsu, Ming-Huang Chen, Chung-Wai Shiau, Ling-Ming Tseng, Kuen-Feng Chen

**Present:** Due to an error during figure assembly, the center panel of Figure 2B was prepared using the wrong set of blots.

Corrected: The corrected Figure 2 is shown below. The authors sincerely apologize for this oversight.

Original article: Oncotarget. 2016; 7(8):9135-49. doi: 10.18632/oncotarget.7035.

**Figure 2:**
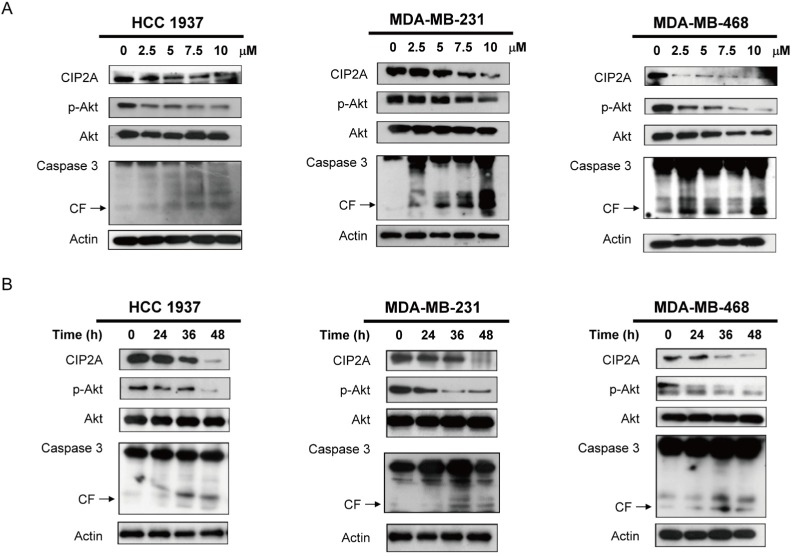
Lapatinib induces apoptosis in association with downregulation of CIP2A and p-Akt in TNBC cells A. Doseescalation effects of lapatinib on CIP2A, p-Akt, and caspase 3 cleavage. Cells were exposed to lapatinib at the indicated doses for 48 hours. B. time-dependent analysis of CIP2A, p-Akt, and caspase 3 cleavage. Cells were exposed to lapatinib (10 μM) for 24, 36 and 48 hours. Cell lysates were prepared and assayed for these molecules by western blotting. Data are representative of three independent experiments. Apoptotic cells were determined by flow cytometry (sub-G1 analysis of propidium iodide-stained cells).

