# Supplementary material for: Lapatinib inhibits CIP2A/PP2A/p-Akt signaling and induces apoptosis in triple negative breast cancer cells
Source: Oncotarget. 2016 Jan 27;7(8):9135–49. doi: 10.18632/oncotarget.7035 (PMC4891031; doi:10.18632/oncotarget.7035)
Supplement: Supplementary file 1 [file oncotarget-07-9135-s001.pdf]

Lapatinib inhibits CIP2A/PP2A/p-Akt signaling and induces apoptosis in triple negative breast cancer cells

Supplementary Material and Methods.

Senescence-associated β-Gal assays

A known marker under the cellular senescence process was appearance of senescence-associated β-galactosidase (SA-β-gal) activity in cancer cells. Briefly, MDA-MB-231 cells plated in 6-well plates were treated with the indicate lapatinib concentrations or DMSO (under 0.01%) for 24h. Cells were then fixed for 10-15 minutes and stained at least for 4 hours at 37C. SA-β-gal activity was revealed with cellular senescence assay kit (Chemicon International Inc., MA, USA) according to the manufacturer's instructions. The SA-β-gal positive cells were counted in random three fields under light microscopy.

Colony Forming Assay

Briefly, MDA-MB-231 cells (2000 cells per dish) plated in 6-cm dishes. Next day, lapatinib 0.1 μM, 1 μM, 5 μM or DMSO was added to the dishes for 24h. After treatment, wash out the medium contain with lapatinib or DMSO, cells then incubated with 10% fresh medium for 7 days allowed to form colonies stained with 0.2% violet and photographed.

Immunohistochemistry (IHC)

Mice xenografts were excised and prepared in paraffin-embedded tissue sections. The process of deparaffinization and antigen retrieval was as previously described [1]. Tissue sections were then treated with 3% hydrogen peroxide for 10 min, blocked with 3% bovine serum albumin (Sigma-Aldrich, MO, USA) and 10% FBS for 90 min, and incubated with anti-p-Akt1/2/3 antibodies (1:200) (Santa Cruz), anti-Ki-67 antibodies (1:1000) (Genemed, Berkshire, UK), and anti-CIP2A antibodies (1:100) (Abcam, Cambridge, UK) overnight at 4°C. All sections were counterstained with hematoxylin.

The Cancer Genome Atlas (TCGA) Data Description

Patient tumor subtypes and gene expression data for breast cancers was accessed from TCGA public data portal (https://tcga-data.nci.nih.gov/tcga/). Level 3 released gene level expression data for expression data for RNAseq were downloaded. The data processing and quality control were done by Broad Institute’s TCGA workgroup. The RNAseq gene expression level 3 data contains Reads per Kilobase per Million mapped reads (RPKM) [2]. RPKM is a widely used RNAseq normalization method, and is computed as follows: RPKM = 109(C/NL), where C is the number of reads mapped to the gene, N is the total number of reads mapped to all genes, and L is the

length of the gene. RPKM values of CIP2A (gene name KIAA1524) and Elk1 gene expression were retrieved and analyzed according to adjacent normal tissue or tumor tissue and according to breast cancer subtypes.

Reference

1. Liu CY, Hung MH, Wang DS, Chu PY, Su JC, Teng TH, Huang CT, Chao TT, Wang CY, Shiau CW, Tseng LM and Chen KF. Tamoxifen induces apoptosis through cancerous inhibitor of protein phosphatase 2A-dependent phospho-Akt inactivation in estrogen receptor-negative human breast cancer cells. Breast cancer research : BCR. 2014; 16:431.
2. Mortazavi A, Williams BA, McCue K, Schaeffer L and Wold B. Mapping and quantifying mammalian transcriptomes by RNA-Seq. Nature methods. 2008; 5:621-628.

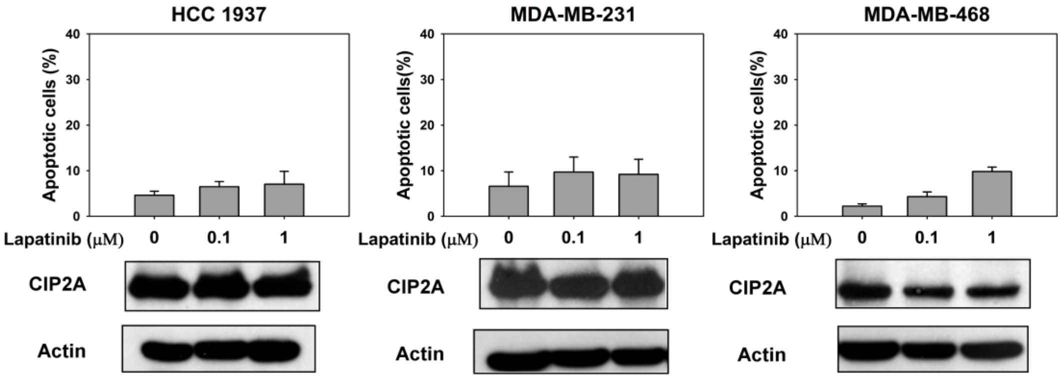

Supplement Figure S1.

Effect of lower loses (0.1 and 1 μM) of lapatinib on CIP2A and apoptosis in TNBC cell lines (HCC-1937, MDA-MB-231, MDA-MB-468). Apoptosis was assessed by flow cytometric subG1 analysis.

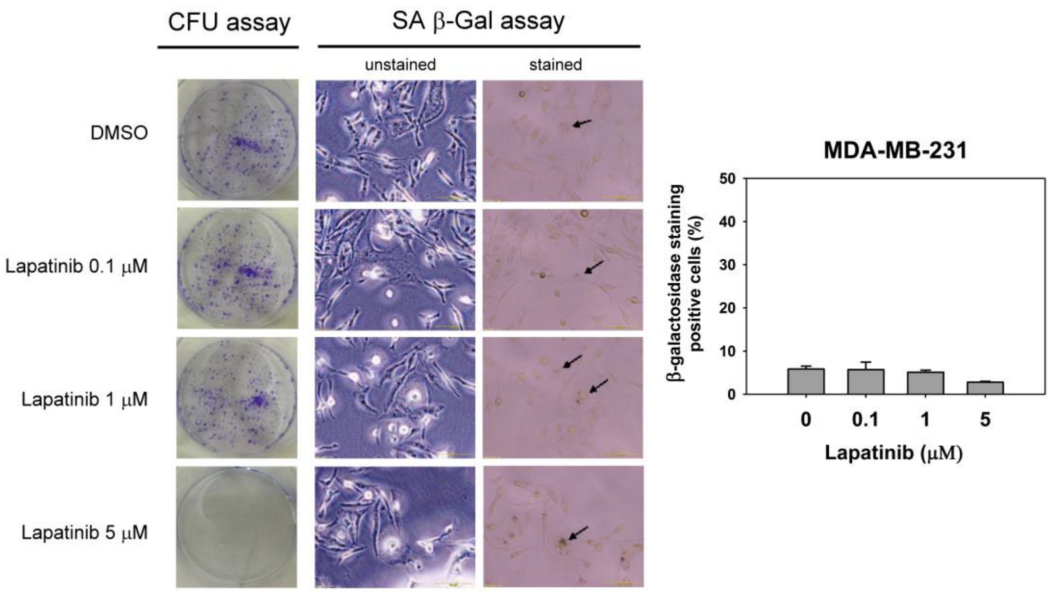

Supplement Figure S2.

Effects of lapatinib on colony forming ability (CFU assay) and senescent-phenotype (SA β-gal assay) in MDA-MB-231 cells. Senescence-associated β-galactosidase (SA β-gal) is a known marker for cell senescence.

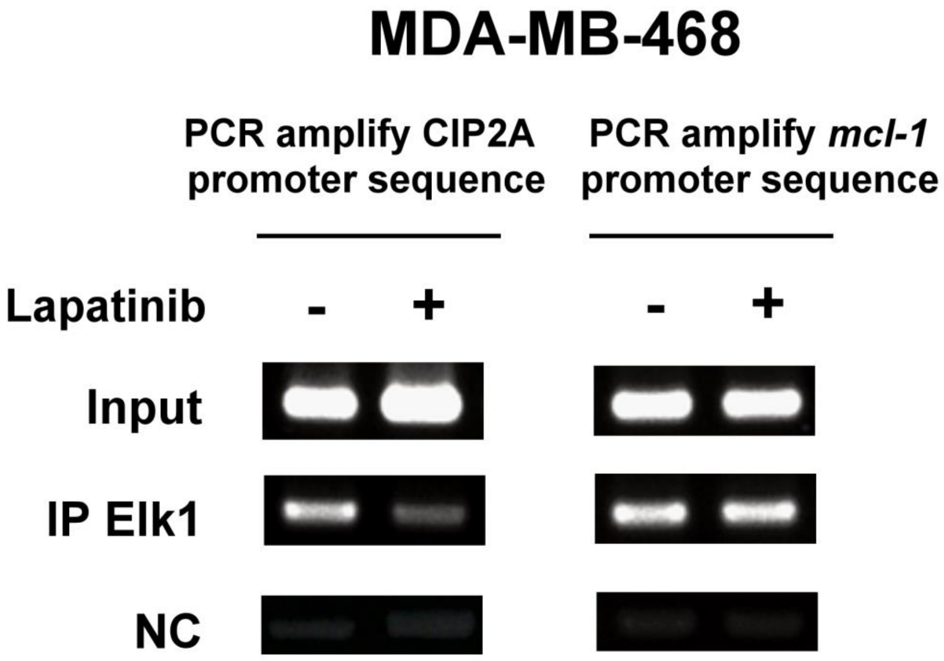

Supplement Figure S3.

Chromatin immunoprecipitation (ChIP) with Elk1 protein and assayed for bound Mcl-1 and CIP2A promoter DNA fragments. NC, negative control using Immunoglobulin G. PCR, polymerase chain reaction.

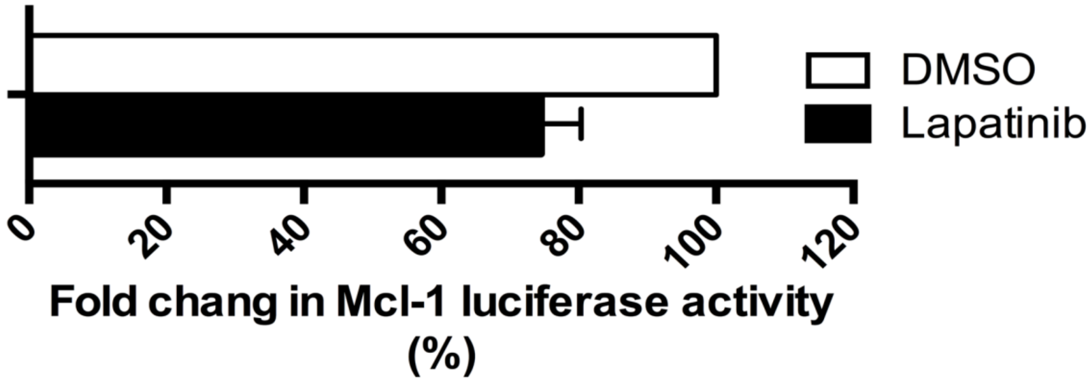

Supplement Figure S4.

Mcl-1 luciferase promoter activity upon lapatinib treatment. MDA-MB-468 cells were transfected for 24 hours with pGL2-*Mcl-1*-promoter and then treated for 24 hours with 5 μM tamoxifen or DMSO. Cell lysates were then assayed for dual-luciferase activity as described in the Methods section. Columns, mean values (n = 3); bars, SD. pGL2-*Mcl-1* was obtained from Addgene (Addgene plasmid # 19132)

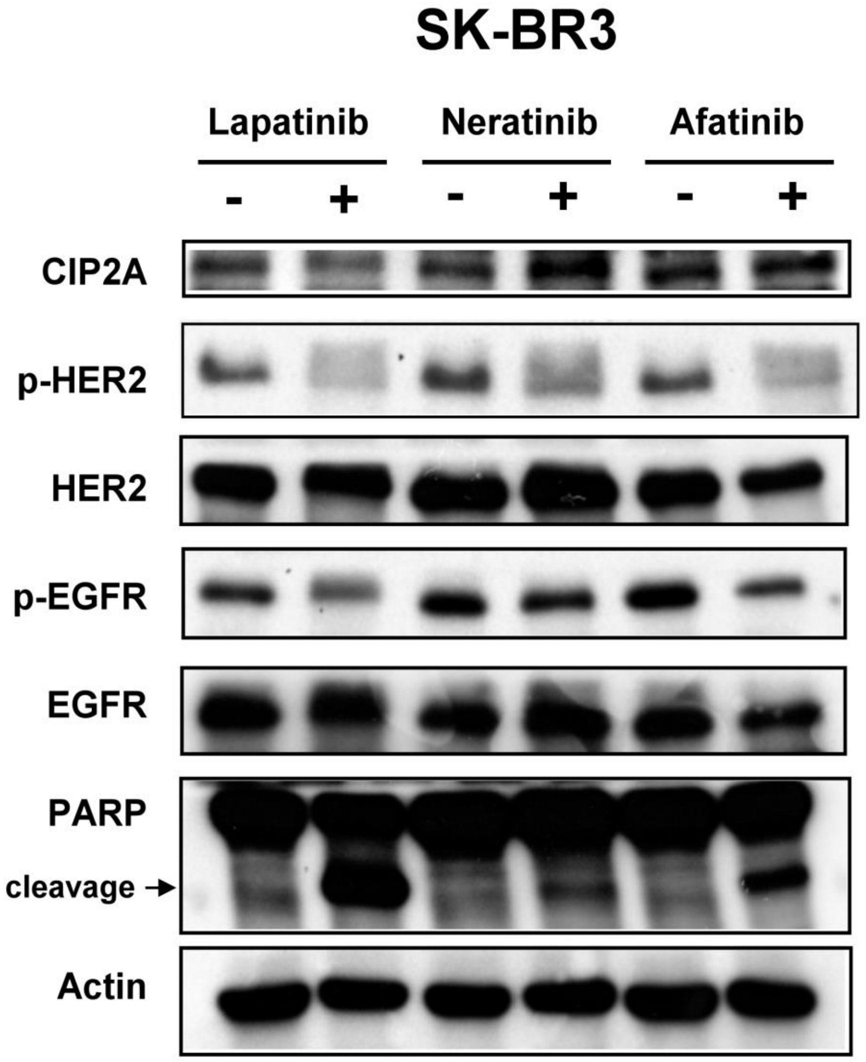

Supplement Figure S5.

Effects of lapatinib, neratinib, and afatinib on CIP2A, p-HER2 and p-EGFR in SKBR-3 cells. Cells were treated with lapatinib (5 μM), neratinib (5 nM), and afatinib (2.5 μM) for 24 h. Cell lysates were prepared and assayed for these molecules by western blotting.

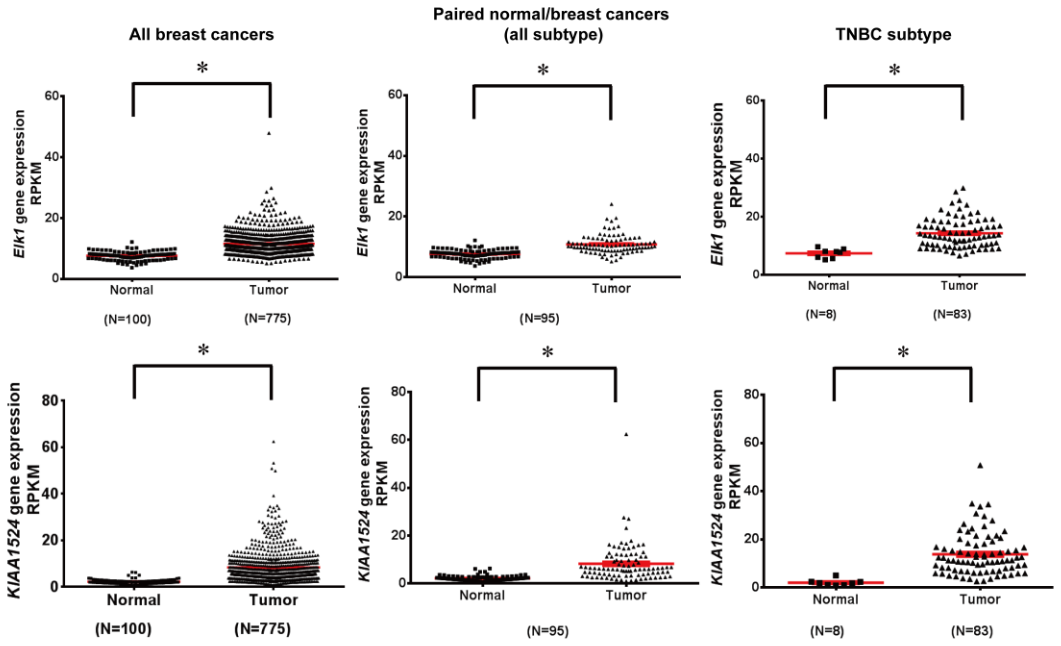

Supplement Figure S6.

Gene expression data of *Elk1* and CIP2A (gene name *KIAA1524*) from TCGA public data portal (https://tcga-data.nci.nih.gov/tcga/). RPKM values of *KIAA1524* and *Elk1* gene expression were retrieved and analyzed according to adjacent normal tissue or tumor tissue and according to breast cancer subtypes (focus on TNBC). * $P<0.05$.
